# CANCER HISTORY AND FRAILTY LEVEL IN OLDER ADULTS AND THEIR HEALTHCARE UTILIZATION: EVIDENCE FROM THE NHIS IN THE US

**DOI:** 10.1093/geroni/igad104.3207

**Published:** 2023-12-21

**Authors:** Mingzhu Su, Siqi Liu, Jiahui Lao, Li Liu

**Affiliations:** Shandong University, Jinan, Shandong, China (People’s Republic); Chinese Academy of Medical Sciences & Peking Union Medical College, Beijing, Beijing, China (People’s Republic); Shandong First Medical University & Shandong Provincial Qianfoshan Hospital, Jinan, Shandong, Jinan, Shandong, China (People’s Republic); Shandong University, Jinan, Shandong, China (People’s Republic)

## Abstract

Previous studies didn’t compare effect measures of frailty between cancer survivors and counterparts without a cancer history, and this makes us unable to judge whether the impact of frailty is more substantial in cancer survivors. This study aimed to examine the association between cancer history and frailty level with healthcare utilization among older adults. A descriptive survey design consistent with the STROBE guidelines was used. 14,562 old adults were identified in the 2019 and 2020 National Health Interview Survey (stratified by old cancer survivors [N=3,944] and older adults without a cancer history [N=10,618]). Frailty was assessed by Fatigue, Resistance, Ambulation, Illness, and Low BMI. Healthcare utilization was measured by urgent care, emergency care, hospitalization, delayed care, and needed but did not get care. Multivariable logistic regressions were used to examine the association between frailty-cancer characteristics and healthcare utilization. Participants with cancer (vs without) were older (75+: 49.7% vs 36.8%) and more likely to be frail (23.8% vs 14.5%) and pre-frail (36.0% vs 33.7%). The strength of associations between cancer and health utilization (emergency care and hospitalization) among older adults was significantly modified by their frailty status. Compared with robust older adults, frail cancer survivors were more likely to report emergency care (OR=4.7, 95% CI: 3.9–5.8) and hospitalization (OR=5.6, 95% CI: 4.5–7.0). Our findings are essential for risk stratification and informed decision-making in future healthcare service provision planning.

